# Modulation of Gut Microbiota Through Dietary Fibers to Enhance Regulatory T Cell-Based Immunotherapy in GVHD Following Hematopoietic Stem Cell Transplantation

**DOI:** 10.3390/nu18081216

**Published:** 2026-04-12

**Authors:** Melika Asayesh, Ata Nazarzadeh, Sanaz Jamshidi, Shayan Keramat, Ireneusz Ryszkiel, Agata Stanek

**Affiliations:** 1 Student Research Committee, Hamedan University of Medical Science, Hamadan 65319, Iran; meli.asa0898@gmail.com; 2 Department of Hematology, Mashhad University of Medical Sciences, Mashhad 9177948974, Iran; atanazar4@gmail.com; 3 Department of Hematology, Faculty of Medical Sciences, Tarbiat Modares University, Tehran 1411713116, Iran; s19n93z@gmail.com; 4 VAS-European Independent Foundation in Angiology/Vascular Medicine, Via GB Grassi 74, 20157 Milan, Italy; shayan.sk1993@gmail.com; 5 Support Association of Patients of Buerger’s Disease, Buerger’s Disease NGO, Mashhad 9183785195, Iran; 6 Department of Internal Medicine, Metabolic Diseases and Angiology, Faculty of Health Sciences in Katowice, Medical University of Silesia, Ziołowa 45/47, 40-635 Katowice, Poland; ryszkielirek@gmail.com

**Keywords:** graft-versus-host disease, allogenic hematopoietic stem cell transplantation, dietary fiber, intestinal microbiota, regulatory T cells, short-chain fatty acids

## Abstract

Graft-versus-host disease (GVHD) is one of the principal complications seen in the recipients of allogenic hematopoietic stem cell transplantation (allo-HSCT), and persists as a leading cause of post-transplant morbidity and mortality. Increasing evidence highlights the crucial influence of the gut microbiome (GM) on transplant outcomes. Microbial dysbiosis, characterized by reduced bacterial diversity and pathogenic overgrowth, is strongly associated with higher rates of complications and mortality. Patients with lower microbial diversity exhibit poorer overall survival (OS) and an increased incidence of acute GVHD (aGVHD). Conversely, restoration of beneficial commensal communities has been shown to enhance immune homeostasis, mitigate GVHD severity, and decrease infection risk. Emerging therapeutic strategies now focus on modulating the intestinal microbiome through dietary interventions, probiotics, prebiotics, and fecal microbiota transplantation (FMT). It has been demonstrated that bacterial metabolites, such as short-chain fatty acids (SCFAs) from the diet, especially a diet rich in fibers, reduce the occurrence/severity of GVHD by inducing regulatory T cells (Tregs), which release anti-inflammatory cytokines and regulate the host immune system. Hence, the implementation of dietary fibers (DFs) could increase beneficial commensals, Treg induction, and improve outcomes such as GVHD and OS in recipients of allo-HCT. Hereupon, this review addresses how a fiber-rich diet modulates GM composition, reinforces epithelial barrier integrity, and improves the efficacy of Treg-based immunotherapy by stabilizing their regulatory phenotype and increasing their functional persistence, ultimately leading to a reduction in GI complications associated with GVHD. Unlike prior reviews that primarily cover the microbiome–GVHD axis or Treg therapies in isolation, this review emphasizes fermentable dietary fibers as a mechanistically grounded, clinically actionable strategy to support Treg stability and persistence via microbiota-derived metabolites. We integrate mechanistic evidence with emerging clinical feasibility data and ongoing trials of prebiotic supplementation in allogeneic HSCT.

## 1. Introduction

Allogeneic hematopoietic stem cell transplantation (allo-HSCT) represents a cornerstone immunotherapeutic strategy and a potentially curative option for a wide range of hematological malignancies and immune disorders. Despite its clinical efficacy, graft-versus-host disease (GVHD) remains a major complication, significantly contributing to post-transplant morbidity and mortality. GVHD arises when donor-derived immune cells recognize recipient tissues as foreign, leading to multi-organ involvement, particularly affecting the gastrointestinal tract, liver, and skin [1,2,3].

The pathophysiology of GVHD is complex and involves interactions between donor immune cells, host tissues, inflammatory cytokines, and the intestinal microenvironment [4,5,6,7].

The gut microbiome (GM) has emerged as a critical determinant of transplantation outcomes. Disruption of microbial homeostasis, characterized by reduced diversity and expansion of pathogenic taxa, is strongly associated with increased GVHD incidence and reduced overall survival [8,9,10,11]. Additional studies further support the role of microbiota composition and diversity in shaping immune responses and clinical outcomes following allo-HSCT [12,13,14,15]. Conversely, preservation or restoration of commensal microbial communities has been linked to improved immune regulation, reduced inflammation, and enhanced epithelial barrier integrity [8,9,10,11].

Recent studies highlight the central role of microbiota-derived metabolites, particularly short-chain fatty acids (SCFAs), in modulating immune responses [12,13,14,15]. These metabolites influence T cell differentiation, promote regulatory T cell (Treg) expansion, and contribute to intestinal homeostasis through both local and systemic mechanisms [12,13,14,15].

Importantly, dietary factors—especially fiber intake—play a key role in shaping microbial composition and metabolic output, thereby influencing host–microbiome interactions and immune regulation [16,17,18].

While previous reviews have addressed the microbiome–GVHD axis and Treg-based immunotherapy separately, the integration of dietary fiber-driven microbiome modulation with Treg stability remains insufficiently explored.

In this review, we provide a mechanistic and translational synthesis linking dietary fiber fermentation, microbiome-derived metabolites, and Treg biology. We further discuss emerging microbiome-targeted therapeutic strategies—including prebiotics, probiotics, fecal microbiota transplantation, and SCFA supplementation—as adjunct approaches to enhance Treg-based immunotherapy in GVHD.

## 2. Literature Search Strategy and Study Selection

This review was conducted as a structured narrative synthesis of the current literature focusing on the role of dietary fiber, gut microbiota, and Treg-mediated immune regulation in the context of HSCT and GVHD.

A structured literature search was performed using PubMed, Scopus, and Web of Science databases to identify relevant studies published in English up to 2025. The search strategy included combinations of the following keywords: “gut microbiome”, “dietary fiber”, “short-chain fatty acids”, “Treg”, “graft-versus-host disease”, and “hematopoietic stem cell transplantation”.

Studies were selected based on relevance to the topic, with priority given to (i) mechanistic studies elucidating microbiome–immune interactions, (ii) preclinical studies investigating SCFA and Treg biology, and (iii) clinical studies conducted in HSCT patients. Reviews and meta-analyses were also included to provide contextual interpretation.

Exclusion criteria comprised studies lacking relevance to HSCT or GVHD, non-peer-reviewed reports, and publications without clear mechanistic or clinical contribution.

For clarity, the included evidence was conceptually grouped into three categories: (i) mechanistic insights (cellular and molecular pathways), (ii) preclinical models, and (iii) clinical evidence in HSCT patients. This framework was used to structure the manuscript and to distinguish between levels of evidence, allowing more consistent interpretation of findings across studies. Given the narrative nature of this review, the search and selection process was not intended to be exhaustive but rather to capture the most relevant and representative evidence.

## 3. Impact of Dietary Fibers on Gut Microbiome Metabolism and Composition

DF has received scientific interest for its direct and indirect beneficial effects on human health, especially playing a critical role in modulating the human digestive system and intestinal health [19]. They constitute the primary energy source for gut bacteria growth, and it has a substantial impact on the composition of the intestinal microbiota and its metabolites [20]. Therefore, a deeper understanding of the interaction between DF and the GM could provide a promising strategy to maintain or improve the microbiota, especially when dysbiosis occurs.

### 3.1. Types of Fibers and Dietary Sources

A comprehensive definition of dietary fiber (DF) was adopted by the Codex Alimentarius, which describes DF as oligosaccharides and other resistant carbohydrates composed of 3–9 monomeric units that are resistant to enzymatic hydrolysis by endogenous enzymes and are not absorbed in the small intestine [21]. DF can be classified in various ways; however, the most common classification is based on water solubility, dividing DF into soluble dietary fiber (SDF) and insoluble dietary fiber (IDF), which differ in their physicochemical properties and physiological effects.

SDFs are generally regarded as fermentable, whereas IDFs are only minimally fermented [22]. Insoluble fibers, such as cellulose and hemicellulose, are found in foods including wheat flour, brown rice, and vegetables. They primarily exert bulking effects and contribute to intestinal transit, while also serving as substrates for partial microbial fermentation. In contrast, soluble fibers, including β-glucan, guar gum, pectin, psyllium, and inulin, are found in whole grains (wheat, oats), legumes, and seeds/nuts [23,24]. SDFs dissolve in water to form viscous gels, which slow gastric emptying and contribute to improved glycemic control and reduced cholesterol levels [25].

Prebiotics, also known as microbiota-accessible carbohydrates (MACs), represent a major fraction of fermentable SDFs and serve as a primary energy source for colonic bacteria, thereby promoting the growth of beneficial gut microbes and supporting host health [26]. DF is fermented and degraded to short-chain fatty acids (SCFAs) in the colon through a complex enzymatic process involving multiple microbial species [19]. The predominant SCFAs—acetate, propionate, and butyrate—are typically present in an approximate ratio of 3:1:1. Acetate is the most abundant SCFA and is produced by a broad range of gut bacteria, including *Bifidobacterium* species, and serves as a substrate for peripheral metabolism. Propionate is mainly produced by *Bacteroidetes* and contributes to gluconeogenesis and immune regulation. Butyrate, primarily generated by Firmicutes such as *Faecalibacterium prausnitzii*, *Roseburia*, and *Clostridia clusters* IV and XIVa, is the main energy source for colonocytes and exerts strong anti-inflammatory and barrier-protective effects. Importantly, the extent of SCFA production depends on the availability of fermentable dietary fiber and the composition of the gut microbiota. Alterations in SCFA profiles, including reduced butyrate levels or shifts in relative proportions, are associated with dysbiosis and increased susceptibility to inflammatory conditions, including GVHD [23,27].

Epidemiological studies have shown that diets rich in fruits and vegetables—key sources of dietary fiber and polyphenols—are associated with reduced cancer-related morbidity and mortality, highlighting the protective role of plant-based dietary patterns in cancer outcomes [28]. Among dietary patterns, the Mediterranean diet (MD), characterized by a high intake of plant-based foods and higher fiber consumption compared to the Western diet (WD), is associated with improved gut microbiota composition, lower cholesterol levels, and increased SCFA production [29,30].

In contrast, the Western diet, typically low in MACs and rich in saturated fats and simple sugars, promotes an unfavorable microbiota composition, often characterized by an increased Firmicutes-to-Bacteroidetes ratio. This imbalance is associated with dysbiosis and increased levels of lipopolysaccharides (LPS), whereas adherence to the MD has been shown to restore microbial balance, enhance beneficial bacteria, and improve metabolic outputs [31,32].

Notably, dietary fibers are not a homogeneous group, and their biological effects vary substantially depending on their physicochemical properties, including solubility, fermentability, and molecular structure. Soluble and highly fermentable fibers, such as inulin and pectin, are more directly associated with SCFA production and immunomodulation [19,23], whereas insoluble fibers primarily contribute to mechanical and barrier-supporting functions [22,26].

This heterogeneity should be considered when interpreting study outcomes, as different fiber types may exert distinct and sometimes non-overlapping effects on the microbiota and immune system.

### 3.2. Effects on Gut Microbiota Composition and Metabolism

The concept of a “healthy microbiota” remains incompletely defined; however, certain bacterial taxa, such as *Lactobacillus* and *Bifidobacterium*, are generally regarded as beneficial, whereas species such as *Escherichia coli*, certain *Clostridium* spp., *Enterococcus* spp., and LPS-producing taxa are more commonly associated with disease states [33,34]. Another factor that defines a healthy microbial composition is the presence of key microbial functions, often referred to as the functional core [33].

DF can enhance beneficial bacteria and inhibit pathogens through several mechanisms, including stimulation of commensal bacterial growth, reduction in luminal pH via SCFA production, and modulation of the gut-associated immune system to identify and counteract pathogens [35]. SCFAs regulate immune responses by interacting with G-protein–coupled receptors (GPCRs), such as GPR41 (FFAR3) and GPR43 (FFAR2), modulating transcriptional activity and inhibiting histone deacetylases (HDACs). These mechanisms enable SCFAs to modulate immune cell function, thereby reducing inflammation and maintaining gut homeostasis [36,37].

In addition, DF can modify the composition of the gut microbiota by altering intestinal transit time, thereby enhancing the growth of certain bacterial taxa [22]. The composition of the intestinal microbiota is influenced by both the quantity and type of dietary fiber intake [38]. Highly fermentable fibers, such as inulin, significantly promote beneficial gut bacteria, including *Bifidobacterium*, *Lactobacillus*, and *Bacteroides*, while reducing potentially harmful taxa such as *Bilophila* and *Proteobacteria* [39,40].

Inulin lowers luminal pH through SCFA production, which may enhance calcium absorption, and has also been associated with protective effects against metabolic syndrome linked to high-fat diets [41,42]. Pectins have a significant impact on SCFA production, with acetate accounting for approximately 75% of total SCFAs, while propionate production decreases to around 16% [43]. Pectin supports gut barrier integrity by strengthening the mucus layer and modulating immune responses, including macrophage and dendritic cell activity [44].

Heinritz et al. demonstrated that increased levels of acetate and butyrate were positively correlated with higher abundances of colonic *Bifidobacteria* and *Lactobacilli* [45]. Notably, butyrate, a key SCFA, serves as a primary energy substrate for colonocytes and plays a protective role in intestinal homeostasis, including prevention of colitis and colorectal cancer [46]. Both *Bacteroidota* and *Actinobacteria* are key fiber-degrading taxa contributing to gut health and metabolic function. It has been demonstrated that a higher *Bacillota*-to-*Bacteroidota* ratio is associated with a lower incidence of infections and diarrhea [47].

These findings suggest that low-fiber diets may reduce gut microbiota diversity in otherwise healthy individuals, disrupt host–microbiota interactions, and potentially increase the risk of disease. Conversely, high-fiber diets have the potential to beneficially alter microbiota composition and metabolic activity, thereby promoting overall health [48,49].

As summarized in Table 1, DF plays a crucial role in maintaining both overall and intestinal health by modulating gut microbiota composition, improving bowel function, and contributing to metabolic homeostasis.

As summarized in Table 2, different types of dietary fibers exert distinct effects on gut microbiota composition, SCFA production, epithelial barrier integrity, and immune regulation, including Treg differentiation and function.

#### 3.2.1. Immune and Barrier Mechanisms

Gut-associated lymphoid tissues (GALTs), which are continuously exposed to microbial and environmental antigens, represent the body’s largest lymphoid organ, comprising approximately 70% of all immune cells. The mucosal immune system acts as a physical barrier, preventing pathogenic microorganisms and immunogenic components from penetrating the mucosa into the body’s internal environment [67].

Intestinal health depends on several interconnected factors, including dietary components, the structural integrity of the mucosa, the gut microbiota (GM), and the immune system. Maintaining a balanced relationship between the mucosal immune system and commensal microbes is a complex and vital process. The GM and its metabolic products play a key role in regulating immune responses and preserving mucosal homeostasis. However, disturbances in this microbial ecosystem, known as dysbiosis, can trigger or contribute to the development of various diseases [68,69].

SCFAs, especially butyrate, play a vital role in strengthening the intestinal barrier, which serves as the body’s first line of defense against harmful pathogens [22]. Multiple signaling pathways mediated by butyrate have been described. For example, butyrate enhances the expression of tight junction (TJ) proteins, such as ZO-1, ZO-2, cingulin, and β-defensin, in intestinal models [70].

Other studies indicate that dietary butyrate promotes barrier function through HIF-dependent regulation of claudin-1 and modulation of the interleukin-10 receptor α-subunit (IL-10RA), alongside repression of claudin-2, which controls paracellular cation and water flux and contributes to diarrhea via the leak pathway [71,72].

Butyrate-induced M2 macrophages promote the differentiation of mucin-producing goblet cells, whereas inhibition of Wnt secretion or ERK1/2 activation attenuates this beneficial effect. Along with butyrate, acetate and propionate positively influence intestinal epithelial cells (IECs) and immune cells by promoting IL-18 production, which improves epithelial barrier integrity [73] and modulates CD4^+^ T cell subsets, thereby supporting gut immune homeostasis [74].

Thus, SCFAs, particularly butyrate, act as an energy source for IECs, contribute to maintaining intestinal barrier function by upregulating TJ expression, stimulate mucus secretion, and promote M2 macrophage polarization with enhanced LC3-associated phagocytosis (LAP), thereby protecting against invading pathogens and suppressing inflammatory responses [75].

#### 3.2.2. Immunomodulatory Effects of SCFAs

The anti-inflammatory effects of DF are largely mediated through interactions with the GM and microbiota-derived SCFAs [76]. The immunomodulatory effects of SCFAs on immune cells and their recruitment are well established. Acetate, propionate, and butyrate can selectively promote the differentiation of T helper (Th)1 and Th17 effector cells, as well as IL-10–producing regulatory T cells (Tregs), depending on the surrounding cytokine milieu and immune context. These regulatory effects involve HDAC inhibition and enhanced mTOR–S6K signaling [77,78].

Pentanoate, another SCFA, also exerts anti-inflammatory effects in CD4^+^ T cells, characterized by increased IL-10 secretion and reduced IL-17A production. This effect is mediated by increased acetyl-CoA availability for histone acetyltransferase (HAT) activity and concurrent inhibition of HDACs, leading to enhanced histone acetylation and increased accessibility of the IL-10 gene promoter [79,80].

It has been demonstrated that SCFAs, predominantly butyrate, modulate immune responses in the gut and may reduce the occurrence or severity of GVHD by inducing Tregs in patients undergoing allo-HSCT [81].

SCFAs also promote the differentiation of B cells into plasma cells and enhance immunoglobulin class switching, leading to increased IgA production via augmentation of the acetyl-CoA pool [82].

In neutrophils, SCFAs enhance innate immune responses by activating FFAR2 and promoting inflammasome assembly, resulting in IL-1β release, which is essential for host defense against pathogens [83]. At the same time, SCFAs can limit excessive nitric oxide (NO) production, thereby contributing to immune regulation [84].

SCFAs also modulate leukocyte recruitment by influencing adhesion molecule expression and chemokine secretion. For instance, SCFAs enhance neutrophil migration through activation of GPR43, mitogen-activated protein kinase (MAPK) signaling, and protein kinase B (PKB) [85].

In individuals with inflammatory bowel disease (IBD), the anti-inflammatory effects of butyrate may be diminished; however, dietary strategies aimed at increasing butyrate production may help alleviate disease severity [86,87].

**Figure 1:**
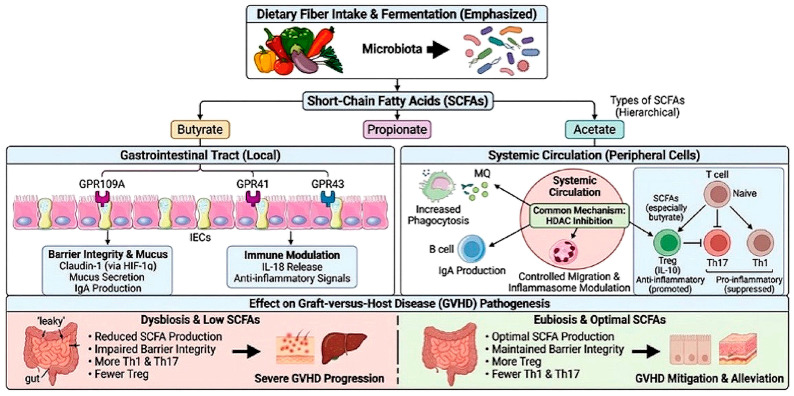
Dietary fiber–derived SCFAs modulate intestinal and systemic immune responses in GVHD. Dietary fiber is fermented by the gut microbiota into SCFAs, including acetate, propionate, and butyrate, which act as key mediators of host–microbiota interactions. SCFAs exert their effects through G-protein-coupled receptors (GPR41, GPR43, and GPR109A) and HDAC inhibition. At the intestinal level, SCFAs enhance epithelial barrier integrity by promoting tight junction assembly, mucus production, and IL-18 secretion, thereby reducing intestinal permeability and microbial translocation. In the systemic circulation, SCFAs regulate immune responses by promoting Treg differentiation and anti-inflammatory signaling, while suppressing pro-inflammatory Th1 and Th17 responses. SCFAs also modulate innate and adaptive immune cells, including macrophages MQ and B cells, contributing to IgA production and overall immune homeostasis. Reduced SCFA levels associated with dysbiosis lead to impaired barrier function and exacerbated inflammation, promoting GVHD progression. In contrast, adequate SCFA production supports immune balance and attenuates GVHD severity. SCFAs, short-chain fatty acids; GPR, G-protein-coupled receptor; HDAC, histone deacetylase; L-18, interleukin-18; Treg, regulatory T cells; Th1/Th17, T helper cell subsets; MQ, macrophages; IgA, immunoglobulin A; GVHD, graft-versus-host disease.

Accordingly, SCFAs may suppress chronic inflammatory responses in innate immune cells through epigenetic regulation, metabolic reprogramming, modulation of cytokine production, and inhibition of pro-inflammatory signaling pathways within the gastrointestinal tract. These mechanisms are schematically illustrated in Figure 1.

It is important to distinguish between association and causality in microbiome research. While numerous studies report correlations between microbial composition, diversity, and metabolic outputs such as SCFA production [33,34,35,48,49], direct causal relationships remain difficult to establish.

Many findings are derived from observational studies or preclinical models, and interventional evidence in humans remains limited. Confounding factors, including diet heterogeneity, antibiotic exposure, and inter-individual microbiome variability, further complicate interpretation.

## 4. Role of Microbiome in Shaping and Modulating Regulatory T Cells

Over the past three decades, Treg cells expressing the transcription factor forkhead box P3 (Foxp3) have been identified as key regulators of immune responses and inflammation, playing a crucial role in maintaining peripheral immune tolerance [88]. Tregs are characterized by the expression of CD25 and Foxp3 and are primarily known for their immunosuppressive functions.

A significant proportion of the Treg population develops in the thymus, where they are referred to as thymus-derived Treg cells (tTregs). However, Tregs can also originate from naïve T cells in peripheral tissues upon exposure to transforming growth factor beta (TGF-β), giving rise to peripherally derived Treg cells (pTregs) [89]. While tTregs and pTregs share similar phenotypic and functional characteristics, they differ in their epigenetic landscapes and transcriptional profiles [90].

The generation of pTregs occurs predominantly in gut-associated lymphoid tissues, particularly within mesenteric lymph nodes (mLNs) and celiac lymph nodes (celLNs) [91]. Organs such as the intestine, which are continuously exposed to diverse foreign antigens, are enriched in peripherally induced Tregs rather than thymus-derived populations [92]. It is now well established that the colon represents the primary site for pTreg development, generating a substantial population of Tregs with a distinct T cell receptor (TCR) repertoire essential for maintaining intestinal homeostasis [93].

Colon-resident Tregs (cTregs) require the presence of microbiota for their development, functional specialization, and maintenance [94]. The first evidence supporting the role of microbiota in pTreg induction was obtained from germ-free (GF) models [95] and antibiotic-treated models [96]. GF mice exhibit a several-fold reduction in Helios^−^ Tregs compared to mice maintained under specific pathogen-free (SPF) conditions [97]. Similarly, antibiotic-induced depletion of microbiota is associated with a marked reduction in Treg frequency [98].

Clostridium species have been shown to promote the induction of Foxp3^+^ Tregs in the colon by stimulating TGF-β production within the lamina propria [99]. However, not all microbial species exert anti-inflammatory effects. For instance, segmented filamentous bacteria (SFB) promote pro-inflammatory Th17 responses and enhance intestinal inflammation [100,101].

At the systemic level, microbiota-derived metabolites, particularly butyrate and propionate, modulate immune responses by promoting the differentiation and function of pTregs [102,103]. Taken together, these findings highlight a dynamic and reciprocal interaction between the gut microbiota and Tregs, suggesting significant therapeutic potential of the microbiome–Treg axis in immune-mediated diseases.

### 4.1. Microbiome-Driven Development of Tregs

Microbial signals, including metabolites and structural components, play a direct role in regulating Treg development, proliferation, and function. These interactions underscore the complex interplay between the microbiota and Tregs, which is essential for maintaining immune homeostasis and preventing inflammatory diseases [104].

Bacterial structural components such as lipopolysaccharides (LPS) and peptidoglycans (PGNs) interact with host immune receptors, including Toll-like receptors (TLRs) and NOD-like receptors (NLRs), thereby shaping immune responses [105,106]. Mazmanian et al. identified polysaccharide A (PSA), a zwitterionic capsular polysaccharide produced by *Bacteroides fragilis*, as a key symbiotic molecule with immunoregulatory properties [107].

PSA interacts with TLR2 on T cells, promoting Treg differentiation and expansion while suppressing Th17 responses [108]. Upon binding to TLR2, PSA induces dendritic cells (DCs) to acquire a tolerogenic phenotype, facilitating the differentiation of naïve CD4^+^ T cells into pTregs in a Gadd45α-dependent manner [109]. This highlights the essential role of DCs in antigen processing and presentation, as well as their ability to shape T cell responses [110].

Additionally, PSA can directly bind to TLR2 on both naïve T cells and Tregs, leading to increased expression of immunoregulatory molecules such as IL-10, TGF-β2, granzyme B, and CCR6 [111]. The clinical relevance of PSA-producing *Bacteroides fragilis* is supported by findings demonstrating reduced abundance of these strains in patients with inflammatory bowel disease (IBD), suggesting therapeutic potential for restoring immune homeostasis [112].

*Bifidobacterium bifidum* strain PRI1 (Bb PRI1) has also been shown to strongly induce pTregs [105]. *Bifidobacterium* species play a crucial role in early-life immune development, particularly in breastfed infants [113]. In germ-free mice mono-colonized with Bb PRI1, this strain promoted the differentiation of CD103^+^CD11b^+^ regulatory DCs. Its cell surface polysaccharides (CSGG) act as TLR2 ligands, inducing DC secretion of IL-10 and TGF-β, thereby promoting an immunoregulatory environment and Treg differentiation. Importantly, these Tregs have been shown to suppress inflammatory colitis in experimental models.

Commensal fungi constitute approximately 2% of the human microbiota [114] and contribute to immune regulation [115]. Dietary fibers such as psyllium and polycarbophil have been shown to increase fecal levels of *Saccharomyces boulardii* [116]. Moreover, fungal dysbiosis has been implicated in IBD pathogenesis [117,118].

Mannan/β-1,6-glucan-containing polysaccharides (MGCP), derived from yeast cell walls, exhibit potent immunomodulatory effects [119]. MGCP promotes Treg differentiation and inhibits Th1 polarization through TLR2-dependent mechanisms. Additionally, MGCP acts via Dectin-1 signaling to induce cyclooxygenase-2 (Cox-2) expression in DCs, promoting a tolerogenic phenotype and enhancing pTreg differentiation.

Microbiota-derived SCFAs act as key signaling molecules between the gut microbiota and the host immune system. They exert their effects through GPCRs such as GPR41, GPR43, GPR109A, and Olfr78, promoting Treg differentiation and expansion [104,120]. GPR41 and GPR43 respond to acetate, propionate, and butyrate, whereas GPR109A is selectively activated by butyrate and niacin [109,121]. These receptors are expressed on both epithelial and immune cells, enabling broad immunomodulatory effects [122].

In dendritic cells, GPR109A signaling promotes IL-10 and retinoic acid production, fostering a tolerogenic environment that supports Treg differentiation [104]. Singh et al. demonstrated that DCs from Gpr109a^−^/^−^ mice fail to induce Treg differentiation in response to butyrate, highlighting the importance of this pathway [121].

SCFAs also regulate Treg differentiation through metabolic reprogramming, as demonstrated in multiple sclerosis patients, where propionate enhances mitochondrial function and Treg suppressive capacity [123]. These findings indicate that SCFAs influence Treg biology through both epigenetic and metabolic mechanisms.

**Figure 2:**
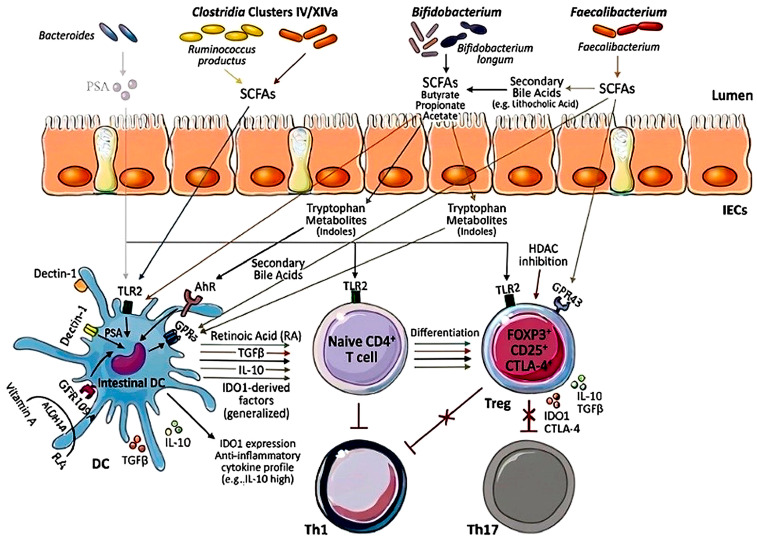
Microbiota-derived metabolites regulate Treg differentiation through epigenetic and metabolic mechanisms. Commensal microbiota produces bioactive metabolites, including SCFAs, secondary bile acids, tryptophan-derived metabolites, and PSA, which modulate host immune responses. These metabolites interact with IECs and immune cells via pattern recognition receptors (e.g., TLR2, Dectin-1) and metabolite-sensing pathways, including GPRs and AhR signaling. At the cellular level, microbial-derived signals condition DCs toward a tolerogenic phenotype characterized by the production of IL-10, TGF-β, and RA, as well as IDO1 activity. These tolerogenic DCs promote the differentiation of naïve CD4^+^ T cells into FOXP3^+^ Treg. SCFAs further regulate Treg differentiation through HDAC inhibition and metabolic reprogramming. In addition to epigenetic effects, SCFAs enhance mitochondrial function and the suppressive capacity of Treg cells, as demonstrated in human studies. These combined mechanisms contribute to the stabilization and function of Treg cells. Functionally, Treg cells suppress pro-inflammatory Th1 and Th17 responses via cytokine-dependent and contact-dependent mechanisms (e.g., IL-10, TGF-β, CTLA-4), thereby promoting immune tolerance. SCFAs, short-chain fatty acids; PSA, polysaccharide A; GPR, G-protein-coupled receptor; AhR, aryl hydrocarbon receptor; HDAC, histone deacetylase; TLR, Toll-like receptor; DC, dendritic cell; RA, retinoic acid; IDO1, indoleamine 2,3-dioxygenase 1; T reg, regulatory T cells; Th1/Th17, T helper cell subsets; IECs, intestinal epithelial cells.

These mechanisms are summarized in Figure 2.

### 4.2. Microbial Regulation of Th17/Treg Balance

Th17 cells are a subset of CD4^+^ T helper cells characterized by the production of IL-17, which plays a key role in promoting tissue inflammation [124]. While our understanding of their roles in both physiological and pathological conditions is still evolving, it is now well established that Th17 cells are essential for protecting mucosal surfaces against microbial pathogens, including bacteria, fungi, and viruses [125].

An altered Th17/Treg ratio has been frequently identified as a hallmark of various metabolic and immunological disorders, including chronic inflammatory diseases [126], autoimmune disorders [127], allergic conditions [99], and malignancies [128]. Accumulating evidence indicates a strong association between the gut microbiota (GM) and the maintenance of Th17/Treg homeostasis. Notably, studies have demonstrated a marked reduction in both colonic Th17 cells and Tregs in germ-free (GF) mice [95,129].

These findings highlight the essential role of microbiota in shaping adaptive immune balance and underscore the importance of microbiome-dependent regulation of T cell subsets. A comprehensive understanding of these interactions is crucial for developing targeted therapies for diseases associated with Th17/Treg imbalance.

Recent studies have consistently demonstrated significant reductions in Th17 and Treg populations in antibiotic-treated or microbiota-depleted animal models [129,130]. Moreover, mice lacking a diverse microbiota exhibit a disrupted Th17/Treg balance compared to those with an intact microbial ecosystem [131].

Segmented filamentous bacteria (SFB), a Clostridia-related species, represent one of the most extensively studied commensals in the context of Th17 immunity [132]. Colonization of GF mice with SFB leads to robust immune activation, including increased IgA secretion, enhanced antimicrobial peptide production, and elevated pro-inflammatory cytokine release in the intestinal lamina propria [100,101,132].

Another key group involved in immune regulation is Clostridia, a diverse and abundant group of Gram-positive, spore-forming bacteria predominantly localized in the cecum and proximal colon [133,134]. Notably, members of Clostridium clusters IV and XIVa play a critical role in promoting colonic Treg differentiation and protecting against inflammatory bowel disease (IBD) [134].

Studies in GF mice have demonstrated that colonization with a defined consortium of 46 Clostridium strains from clusters IV and XIVa induces Helios^−^ colonic Tregs via a MyD88-independent mechanism [95]. This Treg accumulation is largely attributed to microbiota-derived SCFAs, which exhibit potent immunomodulatory and metabolic effects [135].

Collectively, these findings indicate that the balance between Th17 and Treg cells is tightly regulated by microbiota composition and metabolic activity. A deeper understanding of these regulatory mechanisms may facilitate the development of therapeutic strategies aimed at restoring immune homeostasis and correcting Th17/Treg imbalance in disease states.

## 5. Regulatory T Cells and Graft-Versus-Host Disease: Pathophysiology, Immune Dysregulation, and Therapeutic Regulation

Allogeneic hematopoietic stem cell transplantation (allo-HSCT) remains a curative intervention for a range of hematologic malignancies, bone marrow (BM) failure syndromes, and genetic disorders; however, its success is limited by graft-versus-host disease (GVHD), a complex and paradoxical immune reaction in which donor-derived immune cells attack recipient tissues [136,137,138]. Despite advances in HLA matching, conditioning regimens, and immunoprophylaxis, GVHD persists as a leading cause of post-transplant morbidity and mortality [139,140].

The immunological tension between the beneficial graft-versus-leukemia (GVL) effect and detrimental alloreactivity defines the central therapeutic dilemma in HSCT [141,142]. GVHD is not a monolithic process but rather a dynamic interplay of alloimmune recognition, cytokine cascades, tissue-specific damage, and impaired immune tolerance [143,144,145].

Tregs emerge as critical regulators of immune tolerance, capable of suppressing alloreactivity while preserving antitumor responses [146,147]. Thus, understanding Treg biology in GVHD provides both mechanistic insight and translational potential for restoring immune homeostasis through endogenous or therapeutically enhanced regulatory pathways [148,149,150].

### 5.1. Mechanism and Pathophysiology of GVHD

In allo-HSCT, the recipient’s immune system is replaced by donor-derived hematopoietic cells [151]. The therapeutic benefit arises from donor T-cell recognition of residual malignant or infected host cells (GVL effect), yet this same recognition drives GVHD [152,153].

This paradox reflects the dual role of donor lymphocytes, which simultaneously target malignant cells and healthy host tissues [154]. Conditioning regimens induce tissue damage, leading to the release of damage-associated molecular patterns (DAMPs) and microbial products that activate host antigen-presenting cells (APCs) [155,156]. These signals initiate inflammatory cascades and promote T-cell priming, thereby triggering GVHD [157].

GVHD pathophysiology can be viewed as a multi-step process involving tissue injury, host APC activation, donor T-cell priming, and effector-mediated tissue damage. During the initiation phase, host APCs present alloantigens to donor T cells, resulting in their activation and expansion [158,159]. This process is further amplified by pro-inflammatory cytokines such as IL-1β, TNF-α, and IL-6, which promote effector differentiation and cytotoxic responses [160,161]. Subsequently, activated donor T cells migrate to target organs under the control of chemokine receptors including CCR5 and CXCR3, thereby shaping the tissue distribution and severity of GVHD [162,163,164]. The interaction between chemokine receptor expression and local cytokine gradients determines organ-specific involvement in GVHD [163,164].

Acute GVHD (aGVHD), historically defined by onset within 100 days post-transplant, is characterized by epithelial apoptosis, cytotoxic T-cell infiltration, and cytokine-driven inflammation [165]. In contrast, chronic GVHD (cGVHD) manifests as a multi-organ autoimmune-like syndrome involving fibrosis, B-cell activation, and tissue remodeling [166].

Target-organ injury further amplifies disease progression. In tissues with high alloantigen exposure, particularly the intestine, epithelial apoptosis and barrier disruption promote microbial translocation and secondary inflammatory cascades [167,168,169]. In this context, microbiota-derived metabolites, especially SCFAs, may partly counteract epithelial damage and support immune regulation, highlighting the close interaction between barrier integrity, microbial homeostasis, and GVHD pathogenesis [170,171]. Cutaneous and hepatic GVHD manifest clinically as dermatitis and cholestasis, respectively [172,173,174,175]. Ultimately, GVHD reflects a breakdown of immune tolerance and failure of regulatory mechanisms to restrain excessive alloimmune responses [176,177].

### 5.2. Treg Biology: Phenotype, Markers, and Functional Mechanisms

Tregs suppress alloimmunity through both cell–cell contact and secretion of anti-inflammatory cytokines, including IL-10 and TGF-β [140,154]. The central role of IL-2 signaling in Treg biology is underscored by the dependence of Foxp3 expression on STAT5 activation, with IL-2 deficiency leading to impaired Treg function and apoptosis [149].

In GVHD, Tregs preferentially localize to lymphoid organs and target tissues, where they suppress effector T-cell proliferation and promote tissue repair through mediators such as amphiregulin and IL-10 [158,163].

Preclinical and clinical studies demonstrate that adoptive transfer of ex vivo expanded Tregs can prevent both acute and chronic GVHD without impairing the GVL effect [143,178]. This selective immunoregulation allows suppression of pathological inflammation while preserving antitumor immunity [159].

The Th17/Treg ratio represents a dynamic biomarker of disease activity, with Th17 predominance correlating with GVHD severity [140,157].

Treg-mediated suppression is driven by a network of cytokines, including IL-10, TGF-β, and IL-35, which collectively inhibit effector T-cell activation, modulate APC function, and preserve epithelial integrity [141,179,180,181]. Table 3 summarizes the mechanisms and targets of these key immunoregulatory cytokines.

### 5.3. Migration and Localization of Tregs Influenced by Gut Metabolites

Effective control of GVHD requires precise migration of Tregs to inflamed tissues. This process is orchestrated by chemokine receptors and adhesion molecules [155,191].

CCR7 directs naïve Tregs to secondary lymphoid organs, where they suppress initial alloreactive T-cell priming [192]. In contrast, CCR5 and CXCR3 mediate migration toward inflamed GVHD tissues [193].

In intestinal GVHD, homing depends on CCR9–CCL25 and α4β7–MAdCAM-1 interactions, enabling Treg trafficking to the gut lamina propria [194,195]. Once localized, Tregs expressing αEβ7 (CD103) bind E-cadherin on epithelial cells, ensuring retention within inflamed tissues [196].

This spatial positioning allows Tregs to effectively suppress pro-inflammatory Th1 and Th17 responses within GVHD lesions [197]. Tissue-specific migration is also observed in other organs, including CCR4-mediated recruitment in skin and CCR6-dependent homing in the liver [98,198].

In the gut, SCFAs and mucosal Tregs cooperate to maintain barrier integrity and prevent microbial translocation, a key driver of GVHD progression [198,199].

Dietary fiber intake further supports this protective axis, as high-fiber diets enhance epithelial integrity and reduce microbial translocation, whereas fiber deficiency exacerbates barrier dysfunction and disease severity [192,194].

Thus, gut-derived metabolites regulate both the localization and functional stability of Tregs, contributing to immune tolerance restoration.

### 5.4. The Effects of Fiber-Derived SCFAs on the Reduction in GVHD

Both preclinical and clinical observations indicate that SCFA availability is associated with protection against GVHD. Higher SCFA levels, preservation of butyrate-producing bacteria, and receptor expression in the gut correlate with reduced incidence and severity of gastrointestinal GVHD [200]. As described above, SCFAs exert protective effects in GVHD through Treg stabilization and maintenance of epithelial barrier integrity [201].

Observational studies suggest that high-fiber diets and prebiotic interventions support microbiota diversity and butyrate production during conditioning and engraftment, which is associated with improved post-transplant outcomes [202].

Expert analyses highlight SCFAs as diet-responsive mediators capable of modulating immune responses and potentially dissociating GVHD from GVL effects when maintained within physiological ranges [13].

Beyond transplantation, SCFAs demonstrate consistent anti-inflammatory effects across various diseases, supporting their broader role as immune-metabolic regulators [1].

At the tissue level, SCFAs reduce chemokine expression and costimulatory signaling, thereby limiting recruitment and activation of pro-inflammatory effector cells and interrupting the amplification loop of GVHD inflammation [203].

**Figure 3:**
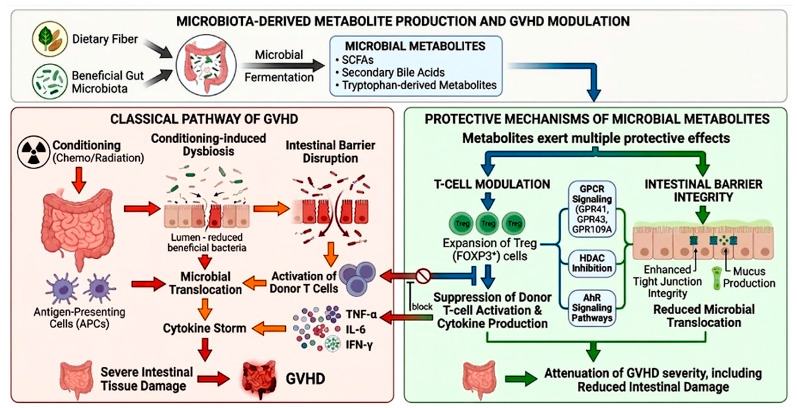
Schematic representation of GVHD pathogenesis and its modulation by dietary fiber–derived microbial metabolites. Dietary fiber undergoes fermentation by the gut microbiota, leading to the production of microbial metabolites, including SCFAs, secondary bile acids, and tryptophan-derived metabolites. On the left, the classical pathway of GVHD development is illustrated: conditioning-induced dysbiosis results in intestinal barrier disruption, promoting microbial translocation and activation of donor T cells. This process triggers a cytokine storm (e.g., TNF-α, IL-6, and IFN-γ), leading to intestinal tissue damage and GVHD. On the right, microbial metabolites exert protective effects through multiple mechanisms, including expansion of Treg (FOXP3^+^), suppression of donor T-cell activation and cytokine production, and enhancement of intestinal barrier integrity (increased tight junction integrity and mucus production, with reduced microbial translocation). These effects are mediated in part via GPCR (GPR41, GPR43, and GPR109A) signaling, HDAC inhibition, and AhR signaling pathways. Collectively, these mechanisms contribute to attenuation of GVHD severity, including reduced intestinal damage. AhR, aryl hydrocarbon receptor; APCs, antigen-presenting cells; FOXP3^+^, forkhead box P3^+^; GPCR, G-protein-coupled receptor; GVHD, graft-versus-host disease; HDAC, histone deacetylase; IFN-γ, interferon gamma; IL-6, interleukin-6; SCFAs, short-chain fatty acids; Treg, regulatory T cells; TNF-α, tumor necrosis factor alpha.

Collectively, the epigenetic and metabolic actions of SCFAs provide a coherent mechanism for reducing GVHD severity while preserving immune competence [204]. These mechanisms are summarized in Figure 3.

Importantly, the translational gap between murine models and human GVHD remains substantial. While preclinical studies consistently demonstrate SCFA-mediated protection through Treg induction and epithelial barrier reinforcement [200,201,202,203], clinical evidence remains limited and largely associative [201,202].

The human microbiome is significantly more complex and influenced by environmental, dietary, and therapeutic factors, which may affect the reproducibility and magnitude of these effects in clinical settings.

## 6. Microbiome and Treg-Mediated Regulation of GVL

The central challenge in allo-HSCT lies in mitigating harmful graft-versus-host disease (GVHD) without compromising the beneficial graft-versus-leukemia (GVL) effect [205,206]. While both processes arise from donor T-cell alloreactivity [207,208], emerging evidence suggests that Tregs can functionally decouple these outcomes by selectively suppressing pathological inflammation while preserving anti-tumor immunity [178,209].

Although both GVHD and GVL are initiated by donor T cells recognizing host alloantigens [210,211], GVL is primarily mediated by CD8^+^ cytotoxic T cells targeting malignant cells [212,213]. Thus, the therapeutic goal is not global immunosuppression but selective modulation of immune responses, enabling control of tissue damage without impairing leukemia clearance [214].

The key mechanistic and functional differences between GVHD and GVL are summarized in Table 4.

Microbiota-derived metabolites, particularly short-chain fatty acids (SCFAs), play a critical role in regulating Treg activity and maintaining the balance between GVHD and GVL, thereby linking diet, microbiome composition, and transplantation outcomes [217]. SCFAs contribute to fine-tuning Treg function and may prevent excessive activation and exhaustion of effector T cells [218].

SCFAs, especially butyrate, promote epigenetic stability of the Foxp3^+^ Treg phenotype, enabling Tregs to maintain their suppressive capacity in the inflammatory post-transplant environment [78,198]. This stabilization enhances resistance to inflammatory signals and sustains Treg-mediated control of alloreactive responses [219,220].

Importantly, Treg-mediated suppression is selective. Tregs primarily inhibit excessive proliferation of alloreactive effector T cells responsible for GVHD, while preserving the cytotoxic machinery required for GVL activity [205,208]. One proposed mechanism underlying this selectivity is the NOTCH1/CD39 axis, in which Tregs suppress conventional T-cell proliferation through NOTCH1 downregulation in peripheral tissues, a major site of GVHD initiation [220,221].

In contrast, anti-leukemia activity is largely maintained within the bone marrow, a compartment relatively less infiltrated by Tregs, allowing cytotoxic T cells to retain their anti-tumor function [211,220]. This spatial compartmentalization represents a key mechanism enabling the dissociation of GVHD from GVL.

Furthermore, different Treg subsets may exhibit distinct functional properties. For example, CD8^+^ Tregs may provide more effective GVHD control while better preserving GVL activity compared to CD4^+^ Tregs, highlighting the importance of Treg heterogeneity in therapeutic strategies [216].

Controlled expansion of Tregs under fiber-rich conditions may further optimize immune balance by limiting excessive activation of effector T cells and preventing immune exhaustion [222].

The functional divergence between T-cell subsets is closely linked to their metabolic programming. Tregs predominantly rely on oxidative phosphorylation (OXPHOS), whereas effector T cells (e.g., Th1 and Th17) depend on aerobic glycolysis [223,224]. SCFAs, particularly butyrate and propionate, support OXPHOS-dependent metabolic fitness in Tregs, enhancing their survival and suppressive function in the post-transplant environment [225,226].

This metabolic advantage enables Tregs to persist and regulate immune responses while simultaneously limiting excessive proliferation of effector T cells, thereby reducing the risk of immune exhaustion [227,228].

Effector T-cell exhaustion, characterized by sustained expression of inhibitory receptors such as programmed cell death protein 1 (PD-1), represents a key mechanism of impaired anti-tumor immunity [229,230]. By dampening excessive inflammation, SCFAs indirectly preserve effector T-cell functionality and sustain the durability of the GVL response [231,232].

Collectively, these findings indicate that microbiome-derived metabolites enable selective immune modulation, in which Treg stabilization suppresses GVHD while preserving GVL activity. This highlights the potential of dietary and microbiome-targeted strategies as adjunctive approaches in allo-HSCT.

## 7. Therapeutic Perspectives of the Fiber–Microbiome–Treg Axis

Although preclinical studies provide compelling mechanistic insights into the fiber–microbiome–Treg axis [205,218], its translation into clinical practice remains challenging. Most available data originate from animal models, and well-designed randomized clinical trials evaluating dietary fiber interventions in GVHD are still lacking [233,234,235,236,237,238,239,240].

Advances in Treg metabolism and microbiome regulation are paving the way for targeted strategies to resolve the GVHD/GVL dichotomy [178,233]. Therapeutic success relies on optimizing Treg dosing and timing [205,208]; current clinical evidence supports the use of genetically engineered Tregs and IL-2/TL1A-Ig fusion proteins to ameliorate GVHD while preserving GVL activity [218,234,235], alongside timed interventions such as delayed donor lymphocyte infusion (DLI) or α-GC administration to avoid early inflammatory toxicity [166,236].

Metabolic conditioning has emerged as a critical optimization strategy [225], where promoting oxidative phosphorylation (OXPHOS) in Tregs—partially mimicking SCFA effects—enhances their suppressive potency and expansion [224,237,238,239].

Selected prebiotic fibers differ in their capacity to modulate microbiota composition, SCFA production, and Treg-mediated immune regulation. As summarized in Table 5, their relative functional potential can be approximated based on available mechanistic and experimental evidence.

Complementary strategies include non-immunosuppressive pharmacologic modulators, such as BET bromodomain inhibitors [205,218], and microbiome-targeted interventions, including fecal microbiota transplantation (FMT), SCFA consortia, and bile acids (BAs), which reinforce immune tolerance without compromising anti-leukemic activity [211,240,241,242,243]. Furthermore, advanced cellular engineering approaches, including chimeric antigen receptor (CAR)-Tregs [244,245,246,247,248] and MSC-derived exosomes [184], offer improved specificity and therapeutic precision.

Collectively, combining metabolic or microbiome-based support with advanced cellular therapies represents a promising and durable strategy to control GVHD while preserving GVL [16].

### 7.1. Adoptive Treg Transfer and Fiber-Enhanced Treg Stability

The translation of Treg biology into clinical practice via adoptive cell therapy (ACT) aims to re-establish immune tolerance in solid organ transplantation (SOT), autoimmune diseases, and GVHD [249,250,251]. Because Tregs comprise only a small fraction of circulating CD4^+^ T cells, Good Manufacturing Practice (GMP) protocols utilize anti-CD3/CD28 stimulation and high-dose IL-2 to generate clinically relevant numbers [249,252,253,254,255].

However, product stability remains a major limitation, as ex vivo expansion can reduce clonal diversity and often fails to ensure complete demethylation of the Foxp3 Treg-specific demethylated region (TSDR), thereby increasing the risk of conversion into pathogenic phenotypes under inflammatory conditions [256,257,258,259,260,261,262].

To mitigate instability and effector overgrowth, expansion protocols frequently incorporate the mTOR inhibitor rapamycin [249,253,255]. Final product validation requires rigorous assessment of suppressive function and epigenetic stability, while cryopreservation remains an additional challenge affecting cell viability and phenotype [249,252].

Emerging evidence strongly suggests that metabolic preconditioning during the ex vivo expansion phase can significantly enhance subsequent in vivo Treg stability and therapeutic efficacy [258]. SCFAs, the primary metabolites of dietary fiber fermentation, have been identified as powerful modulators for optimizing adoptive T cell therapy [258,259].

SCFAs, particularly butyrate and propionate, have been shown to augment the differentiation and function of human induced Tregs in vitro [263]. Studies directly comparing different in vitro induction protocols have confirmed that the addition of butyrate, alongside IL-2 and TGF-β, represents a viable and effective strategy for generating stable human Foxp3^+^ Tregs [255].

Metabolic preconditioning has been further formalized through advanced ex vivo expansion strategies, such as the “3C” protocol (sodium butyrate, UNC0646, and vitamin C), which generates stable, antigen-specific inducible Tregs (iTregs). This approach induces complete demethylation of the Foxp3 conserved non-coding sequence (CNS2) region [264], a process further supported by vitamin C acting as a cofactor for TET enzymes [261,262], ultimately yielding cells that resist inflammatory destabilization and maintain lineage stability [264].

In vivo, systemic SCFAs such as propionate and acetate, derived from high-fiber diets, enhance Treg precursor generation in the bone marrow [17] and induce donor-specific tolerance [265], suggesting that dietary fiber plays a critical role in supporting the persistence and functionality of adoptively transferred Tregs [259,265].

Consequently, translational strategies addressing the dysbiosis–GVHD axis—including low-dose IL-2, microbiome modulation, and engineered Tregs [266,267,268]—rely heavily on metabolic support provided by fermentable dietary fibers to achieve optimal therapeutic efficacy [16,269,270].

This paradigm supports the development of synergistic therapeutic approaches integrating adoptive Treg transfer with targeted dietary or microbiome-based interventions, enabling the establishment of a stable, tolerogenic gut microenvironment [271,272].

### 7.2. Low-Dose IL-2 and Microbiome Synergy

LD IL-2 therapy augments immune regulation in cGVHD by capitalizing on the high-affinity IL-2 receptor (CD25) to drive preferential Foxp3^+^ Treg expansion over T conv [179,273,274,275,276]. Validated in refractory settings [277,278], this approach yields objective responses in over 50% of adults [277] and up to 85% in pediatric cohorts [279,280], particularly when initiated prior to severe fibrosis [277,281].

Durable clinical success directly correlates with normalization of the T conv ratio [277,282,283], driven by up to fivefold expansion of Tregs displaying an activated, suppressive phenotype [273,275,284]. Mechanistically, IL-2 acts as a potent homeostatic cytokine, promoting Treg survival and maintenance via PD-1 upregulation [285,286].

Importantly, despite these encouraging clinical outcomes, the interaction between LD IL-2 therapy and the gut microbiome remains incompletely understood and is supported predominantly by preclinical evidence [287].

Preclinical studies suggest that LD IL-2 therapy can remodel gut microbiota composition, fostering a more tolerogenic GM and ameliorating GVHD [288,289]. This cross-talk is fundamentally metabolic: while IL-2 provides the signal for Treg proliferation, fiber-derived SCFAs provide critical metabolic and epigenetic stabilization required for sustained Treg function at the site of inflammation [78,290].

SCFAs modulate T cell differentiation via activation of the mTOR pathway, promoting Treg differentiation over effector T-cell lineages, particularly under defined cytokine milieus [78]. Thus, a DF-enriched environment may precondition immune cells to respond more effectively to IL-2–mediated tolerance signals, although this concept requires validation in clinical studies [270].

This combined approach using LD IL-2 for numerical expansion and DF for metabolic and epigenetic stabilization represents a promising but still emerging therapeutic strategy [291,292].

The safety of this approach is supported by early clinical data showing that prophylactic use of ultra-low-dose IL-2 (UL-2) expands Tregs without significantly increasing GVHD risk or compromising the GVL effect [293,294].

Clinical protocols combining donor Treg infusions with daily LD IL-2 for steroid-refractory cGVHD yielded approximately 50% response rates, supporting the role of IL-2 as a key homeostatic cytokine sustaining adoptively transferred Tregs rather than a standalone curative therapy [295].

Parallel preclinical studies utilizing IL-2 with rapamycin demonstrated reduced lethal aGVHD through enhanced metabolic conditioning and donor Treg expansion [296,297].

Overall, LD IL-2 therapy represents an important component of Treg-based strategies; however, its full therapeutic potential may depend on integration with microbiome-derived metabolic support, particularly SCFA-generating dietary fibers [270,291].

### 7.3. Engineered CAR-Tregs with Fiber-Driven Metabolic Optimization

The next generation of GVHD therapy will emerge from the deliberate integration of advanced T cell engineering with metabolic support provided by the DF–microbiome axis [266,272,298]. This approach aims to establish highly specific and durable immune tolerance through combined antigen targeting and metabolic conditioning [299].

The cutting edge of Treg therapy involves CAR-Tregs engineered to target alloantigens (e.g., HLA molecules) [300,301]. These “living precision medicines” are designed to suppress GVHD while preserving the beneficial GVL effect [245,302,303]. However, maintaining CAR-Treg function and stability in inflammatory environments remains a major challenge due to tonic signaling and metabolic stress [304,305].

Tregs exhibit distinct metabolic programming, relying primarily on fatty acid oxidation, in contrast to glycolysis-dependent effector T cells [271]. The SCFA butyrate, a product of DF fermentation, reinforces Treg lineage stability [306]. Through HDAC inhibition, butyrate epigenetically stabilizes the Foxp3 locus, enhancing resistance to inflammatory destabilization [78,306].

Future manufacturing strategies may incorporate SCFAs or their analogs during ex vivo expansion to generate metabolically optimized (“Super-Treg”) products with improved persistence and suppressive capacity [307].

The preclinical success of transient mTOR inhibition with rapamycin provides a model for how SCFA-driven HDAC inhibition can be leveraged as a physiological form of metabolic conditioning to enhance Treg stability and function [308,309].

This metabolic “arming” is critical for ensuring that engineered Tregs maintain lineage stability and suppressive function within the inflamed gut microenvironment [310].

In conclusion, the future of GVHD therapy depends on the synergistic integration of cellular engineering and microbiome-derived metabolic support, enabling stabilization and potentiation of Treg function [266,298].

The clinical translation of dietary fiber interventions capable of increasing SCFA production provides a direct mechanistic link supporting the efficacy of advanced cellular therapies in vivo [311,312].

This multi-modal therapeutic strategy, summarized in Table 6, leverages the fiber–microbiome axis to enhance both stability and efficacy of next-generation immunotherapies.

### 7.4. Microbiome-Targeted Therapeutic Strategies in GVHD

In addition to Treg-based cellular therapies, several microbiome-targeted strategies have emerged as potential adjunctive approaches to modulate immune responses and improve outcomes in GVHD. These include prebiotics, probiotics, fecal microbiota transplantation (FMT), and direct supplementation with microbial metabolites such as SCFAs [9,16,202].

Prebiotics, including fermentable dietary fibers such as inulin and oligosaccharides, promote the growth of beneficial commensal bacteria and enhance SCFA production, thereby indirectly supporting Treg differentiation and intestinal barrier integrity [19,23,39].

Probiotics aim to restore microbial balance through the administration of live microorganisms. Although preclinical studies suggest immunomodulatory benefits, clinical data remain limited and heterogeneous, particularly in immunocompromised populations, where safety concerns persist [11,33].

Fecal microbiota transplantation (FMT) has shown promising potential in restoring microbial diversity and improving outcomes in GVHD, particularly in the context of microbiota-targeted interventions [11,240,241,242,243]. However, challenges related to standardization, donor selection, and long-term safety remain to be addressed.

SCFA supplementation, particularly with butyrate and propionate, represents a more targeted strategy aimed at directly modulating immune and epithelial functions. SCFAs have been shown to promote Treg differentiation, enhance epithelial barrier integrity, and reduce inflammatory signaling pathways [13,78,198]. While preclinical data are robust, clinical evidence remains limited.

Collectively, these approaches highlight the translational potential of microbiome modulation as an adjunct to Treg-based immunotherapy; however, further well-designed clinical trials are required to define their efficacy, safety, and optimal integration into therapeutic protocols [233,234,235,236,237,238,239,240].

## 8. Discussion

Allo-HSCT is considered one of the most powerful therapeutic strategies for treating hematologic malignancies and immune-related disorders, and in certain cases represents the only curative treatment option [1,2]. Despite its clinical success, its application is frequently hindered by GVHD, in which donor-derived immune cells target host tissues, particularly the gastrointestinal tract, liver, and skin, leading to significant morbidity and mortality [3,4].

Mechanistically, pre-transplant conditioning with radiation or alkylating agents induces substantial epithelial and tissue injury, resulting in the release of DAMPs. These molecules activate APCs and initiate inflammatory cascades that are central to GVHD pathogenesis [155,156,157]. Within the intestinal tract, apoptosis of crypt cells, depletion of Paneth and goblet cells, and disruption of epithelial barrier integrity promote microbial translocation and amplify systemic inflammation [168,169].

In addition to direct tissue injury, profound dysregulation of the GM—an essential regulator of mucosal and systemic immune homeostasis—further exacerbates immune imbalance and inflammatory responses [4]. In parallel, thymic damage occurring during transplantation impairs central immune tolerance by reducing Treg generation and promoting the expansion of pathogenic effector cells [169,314]. Consequently, a major challenge in allo-HSCT remains achieving a balance between preserving the beneficial GVL effect and preventing GVHD-associated tissue injury [205].

Dietary factors continue to play a key role in shaping microbiome composition in the transplant setting. Consequently, dietary interventions and targeted nutritional strategies are increasingly being explored as modulators of microbiota and immune responses [315,316,317,318,319].

Recent research highlights the growing interest in combining FMT with dietary strategies, particularly fiber-based interventions, to mitigate GVHD severity following HSCT. However, the optimal composition, timing, and frequency of such combined interventions remain insufficiently defined, and current evidence is limited by heterogeneity of study designs and small cohort sizes [320,321]. FMT has demonstrated efficacy in steroid-refractory GI GVHD, achieving partial remission in approximately 74% and complete remission in 50% of cases [322]. Early clinical trials, including NCT03819296, NCT04038619, NCT0421041, and NCT04163289, further support its potential to induce remission and reduce immune-related adverse events [323,324].

While evidence supporting the use of nutrients, prebiotics, and probiotics in GVHD remains limited, FMT currently represents the most clinically validated microbiome-targeted intervention [322,325].

Beyond dietary fiber, several additional strategies may contribute to enhancing gut microbial diversity and functional resilience. These include the use of probiotics, which introduce beneficial microbial strains; postbiotics, defined as microbial-derived bioactive compounds; and synbiotics, combining prebiotics and probiotics to synergistically support microbial growth. In addition, FMT represents a direct approach to restoring microbial diversity, particularly in cases of severe dysbiosis. Emerging interventions also include microbiota-targeted therapies aimed at modulating specific bacterial taxa or metabolic pathways. Collectively, these approaches highlight that modulation of gut microbiota can be achieved through multiple complementary strategies beyond fiber supplementation alone, although their efficacy and safety require further validation in well-designed clinical studies [11,19,240,241,242,243]. Nevertheless, these findings should be interpreted cautiously due to variability in patient populations, intervention protocols, and endpoints across studies.

Dietary factors, particularly fiber and prebiotics, also influence transplant outcomes. A Turkish pediatric study demonstrated associations between pre-transplant dietary patterns and clinical parameters, including neutrophil engraftment and febrile neutropenia [326]. Supplementation with oligosaccharides, glutamine, and dietary fibers reduced mucositis, diarrhea, and weight loss, although effects on GVHD incidence remained modest [327]. These observations suggest supportive benefits but do not establish causality or standardized therapeutic recommendations.

Beyond FMT, DF independently modulates immune responses and represents a key component of immunonutrition. Immunonutrition, defined as the use of nutrition therapy to modulate immune responses [328], has been proposed as a safe adjunctive strategy to help reduce GVHD incidence and infection risk [329,330,331]. Emerging evidence suggests that supplementation with immunonutrition agents may improve clinical outcomes in critically ill patients [332,333,334].

Dietary fibers and prebiotics are metabolized by commensal bacteria, particularly *Clostridium* species (clusters IV and XIVa), generating SCFAs such as butyrate, propionate, and acetate [335,336,337]. These metabolites exert multiple protective effects, as described in earlier sections [78,338,339]. Elevated SCFA levels are associated with enhanced regulatory immune responses. Importantly, not only total SCFA levels but also their relative distribution may influence immune responses, with butyrate exerting the strongest local effects on epithelial integrity and Treg stabilization [78].

Supplementation with prebiotics and resistant fibers, including resistant starch, FOS, and GOS, has been shown to restore microbial homeostasis and increase SCFA production [12,340,341,342]. In murine models, GOS administration reduced GVHD severity and increased butyrate-producing bacteria [12], while combined fiber interventions improved microbial diversity and clinical outcomes [343]. Clinical studies report reduced mucositis and improved survival with combined nutritional interventions [327].

However, it should be emphasized that a substantial proportion of these findings derive from preclinical models, and their direct translation into clinical practice remains uncertain.

This limitation is particularly relevant in the HSCT setting, where human studies remain scarce and are frequently affected by substantial methodological heterogeneity. Differences in conditioning regimens, antibiotic exposure, nutritional support, donor type, graft source, and timing of microbiome sampling markedly complicate comparisons across studies. In addition, the human microbiome is considerably more complex and variable than experimental animal models, which limits direct extrapolation of mechanistic findings. Therefore, although animal studies have been instrumental in identifying SCFA-mediated effects on epithelial integrity and immune regulation, the clinical significance of these mechanisms in HSCT patients should be interpreted with caution until confirmed in well-designed prospective studies.

Importantly, recent literature emphasizes the need for well-designed randomized clinical trials to validate microbiome-targeted and dietary interventions in GVHD, as current evidence remains largely associative and derived from heterogeneous clinical and preclinical datasets [344,345,346].

Diet composition exerts differential immunological effects. High-fat diets promote expansion of pro-inflammatory taxa such as *Bilophila wadsworthia* [347], whereas high-fiber diets enrich beneficial microbial communities and promote Treg differentiation [348].

Importantly, microbiome alterations begin prior to transplantation. Chemotherapy, conditioning regimens, and antibiotic exposure lead to reduced microbial diversity and dysbiosis [349,350,351,352,353,354]. Broad-spectrum antibiotics deplete beneficial taxa such as Clostridia and *Faecalibacterium prausnitzii*, impairing Treg-inducing pathways [322,355,356].

Broad-spectrum antibiotics deplete beneficial taxa such as Clostridia and Faecalibacterium prausnitzii, which normally promote Treg expansion via TGF-β and RA signaling in DCs [322,352]. Importantly, dysbiosis in the context of allo-HSCT is not only characterized by reduced microbial diversity but also by specific taxonomic shifts. Expansion of pathogenic taxa, particularly Enterococcus and Proteobacteria, has been consistently associated with increased GVHD severity and poorer clinical outcomes, whereas a higher abundance of commensal SCFA-producing bacteria, including *Blautia*, *Faecalibacterium*, and members of *Clostridia clusters* IV and XIVa, correlates with improved survival and reduced GVHD incidence [204,357,358,359,360,361,362]. Conversely, expansion of Enterococcus species promotes pro-inflammatory Th17 responses and worsens GVHD [363].

Additional medications, including proton pump inhibitors, antidepressants, and opioids, further disrupt GM composition [363,364]. Combined with reduced oral intake due to mucositis and nausea, these factors exacerbate dysbiosis and intestinal barrier dysfunction [365,366].

Patients with higher microbial α-diversity prior to transplantation—particularly enriched in SCFA-producing taxa such as *Blautia*, *Roseburia*, *Faecalibacterium*, *Bacteroides*, and *Coprococcus*—demonstrate improved OS and better transplant outcomes [204,360,361,362,367,368,369], providing clinical support for the relevance of microbiome-derived metabolites in modulating HSCT outcomes. Conversely, reduced microbial diversity prior to HSCT is associated with poorer survival and higher transplant-related mortality [360]. Therefore, both microbial diversity and taxonomic composition should be considered complementary indicators of microbiome status in HSCT patients.

Although microbiota-targeted interventions in the pre-transplant setting remain limited, available evidence suggests potential benefits. Supplementation with glutamine, DF, oligosaccharides, and resistant starch has been reported to alleviate diarrhea, mucositis, and bacteremia while improving survival rates [327,343,370,371].

Similarly, while probiotic and FMT-based strategies demonstrate promise, their safety, standardization, and long-term clinical effects require confirmation in well-designed randomized trials [372,373,374,375,376].

Collectively, these findings underscore that the integrity of the GM prior to transplantation plays a decisive role in post-transplant outcomes. Early implementation of microbiota-preserving and nutritional strategies may help maintain microbial homeostasis, reduce treatment-induced toxicity, and ultimately enhance transplant success [375,376].

Our findings align with existing evidence suggesting that diets rich in fiber and fermented foods increase microbial diversity, reduce inflammatory markers, and support balanced immune responses [377]. Fiber-enriched enteral nutrition promotes SCFA-producing species and intestinal recovery, whereas fiber-deficient nutrition delays microbiome restoration and alters metabolic pathways [378,379].

Taken together, the available evidence supports a model in which DF enhances the efficacy of Treg-based immunotherapy by stabilizing their regulatory phenotype, increasing their functional persistence, and promoting microbiome-derived metabolite production. However, further well-designed clinical studies are required to establish causality, define optimal dietary strategies, and integrate these approaches into standardized therapeutic protocols.

To further synthesize the complex and multidimensional interactions between the gut microbiome and transplant outcomes, several conceptual and mechanistic models have been proposed. These models integrate microbial composition, metabolic activity, epithelial barrier integrity, and immune regulation to explain their differential impact on GVHD and GVL. The most relevant and widely described models, along with their key mechanisms and clinical implications, are summarized in Table 7.

## 9. Limitations of Current Evidence

Despite the growing body of evidence supporting the role of dietary fiber–microbiome interactions in immune regulation, several limitations must be acknowledged. A substantial proportion of the available data derives from preclinical models, particularly murine studies, which may not fully recapitulate the complexity of human immune responses and microbiota composition.

This limitation is particularly relevant in HSCT research, where human studies remain limited and are frequently affected by significant methodological heterogeneity. Variability in conditioning regimens, antimicrobial exposure, dietary intake, supportive care, and timing of sample collection can substantially influence microbiome composition and metabolite profiles, thereby reducing comparability between studies and limiting causal inference.

Furthermore, most available clinical data are observational, which precludes establishing direct causal relationships between dietary fiber intake, microbiota alterations, and clinical outcomes such as GVHD.

In addition, dietary fiber represents a heterogeneous group of compounds with distinct physicochemical properties and biological effects. Differences in fiber type, dose, and fermentability across studies further complicate the translation of current findings into standardized clinical recommendations.

## 10. Conclusions

In conclusion, the pathogenesis of GVHD following allo-HSCT remains a major clinical challenge, reflecting a complex interplay between conditioning-induced tissue injury, loss of peripheral tolerance, and dysregulated alloreactive immune responses. This review highlights the central role of the GM and its metabolic activity in modulating this process.

The DF–microbiome–Treg axis may represent a promising mechanistic and translational framework linking diet, microbial metabolism, and immune regulation in GVHD. Intake of fermentable DF emerges as a modifiable factor with potential therapeutic relevance, particularly in the post-transplant setting, where dysbiosis, antibiotic exposure, and reduced oral intake disrupt SCFA-producing microbial communities.

Fermentable DFs function not merely as nutrients but as substrates for microbial metabolism, leading to the production of SCFAs, particularly butyrate. These metabolites exert dual protective effects. First, they support intestinal epithelial integrity by serving as an energy source for colonocytes, enhancing tight junction stability, promoting mucus production, and limiting microbial translocation and systemic inflammation.

Second, SCFAs act as epigenetic and metabolic regulators of immune responses, promoting Treg differentiation and stabilizing Foxp3 expression through HDAC inhibition. This mechanism enhances Treg persistence and functional stability in the inflammatory post-transplant environment, thereby limiting pathological alloreactivity while potentially preserving the beneficial GVL effect.

Importantly, although preclinical data consistently support this model, clinical evidence remains limited and largely associative, underscoring the need for cautious interpretation.

The therapeutic targeting of the DF–microbiome–Treg axis—through dietary strategies, microbiome modulation, or metabolic support of cellular therapies—represents a promising approach for restoring immune homeostasis and improving outcomes in allo-HSCT recipients.

Future research should prioritize well-designed, adequately powered prospective clinical trials in HSCT settings to validate microbiome-targeted and dietary interventions, define optimal fiber types and dosing strategies, and establish their integration into standard GVHD prevention and treatment protocols.

## Figures and Tables

**Table 1:** Key studies on dietary fiber, gut microbiota, and immune responses.

Study	Experimental Model	Fiber Type	Main Findings
[50,51,52,53]	Human/experimental studies	Inulin (soluble fiber)	Increases SCFA production; promotes growth of *Bifidobacterium*; reduces body weight, cholesterol, and blood glucose levels
[51,53,54,55]	Human/in vitro	Pectin (soluble fiber)	Modulates gut microbiota composition; enhances SCFA production; improves glucose metabolism; supports beneficial bacteria; induces apoptosis in colon cancer cells
[51,52,53,55,56]	Animal/ human studies	β-glucan (soluble fiber)	Prebiotic effect; increases SCFA production; reduces glucose absorption; exerts anti-inflammatory and immunomodulatory effects; supports *Lactobacillus* and *Bifidobacterium*
[53,57,58,59]	Animal studies	Fructans (soluble fiber)	Enhances immune function; increases *Bifidobacterium*; reduces Bacteroidetes; promotes SCFA production and fecal bulking
[51,52,60]	Human studies	Oligofructose (prebiotic fiber)	Reduces plasma LPS levels; supports microbiota homeostasis; exerts metabolic benefits
[53,57,58,61]	Animal studies	Cellulose (insoluble fiber)	Promotes SCFA production; increases *Akkermansia*; enhances mucus production and goblet cell activity; protective against colitis
[53,57,58,62]	Animal studies	Hemicellulose (insoluble fiber)	Improves microbiota diversity; enhances immune function; supports protection against chronic diseases
[63,64,65,66]	Animal/ human studies	Chitin–chitosan	Reduces body weight and cholesterol; exerts cardioprotective effects; modulates immune responses

SCFAs, short-chain fatty acids; LPS, lipopolysaccharide.

**Table 2:** Functional effects of key dietary fibers on microbiota composition, SCFA production, epithelial barrier integrity, and immune regulation [19,23,26,39,40,43,44].

Fiber Type	Main Microbiota Effect	Dominant SCFAs	Barrier Integrity	Treg/Immune Effect
Inulin (fructans)	↑ *Bifidobacterium*, *Lactobacillus*	Acetate, butyrate	↓ pH, ↑ tight junctions	↑ Tregs, anti-inflammatory
Pectin	↑ *Bacteroides*, microbiota diversity	Acetate (dominant)	↑ mucus layer	Modulates macrophages, DCs
β-glucan	↑ *Lactobacillus*, *Bifidobacterium*	Butyrate	Enhances epithelial repair	Anti-inflammatory, immunomodulatory
Resistant starch	↑ *Faecalibacterium*, *Ruminococcus*	Butyrate (high)	Strong barrier support	↑ Tregs, ↓ Th17
Oligofructose/FOS	↑ *Bifidobacterium*	Acetate, propionate	Indirect barrier support	↓ LPS, immune modulation
Cellulose	↑ diversity (low fermentation)	Low SCFA	Mechanical support	Minimal direct immune effect
Hemicellulose	↑ microbiota diversity	Mixed SCFA	Supports barrier	Anti-inflammatory
Chitin–chitosan	Modulates microbiota composition	Variable	Barrier support	Immunomodulatory

SCFAs, short-chain fatty acids; LPS, lipopolysaccharide; Tregs, regulatory T cells; ↑, increase; ↓, decrease.

**Table 3:** Primary functions, mechanisms of action, and unique characteristics of key soluble mediators (IL-10, TGF-β, and IL-35) produced by Foxp3^+^ regulatory T cells.

Cytokine	Primary Targets	Key Mechanisms of Action	Unique Roles & Effects
IL-10 [141,143,145,178,181,182,183]	APCs, conventional T cells (T conv), Macrophages, B-cells	Reduces major histocompatibility complex (MHC) II and co-stimulatory molecules, Blocks IL-12/IL-23 pathways, preventing Th1/Th17 generation	Broad-spectrum anti-inflammatory; absence of IL-10 signaling significantly exacerbates GVHD pathology
TGF-β [13,176,184,185,186,187]	T conv, Epithelial Tissues, Existing Tregs	Limits effector T-cell expansion and pro-inflammatory cytokines (IFN-γ, IL-17), Converts CD4^+^ T conv into pTregs	Acts as both an immunosuppressant and a tissue-repair agent (restores gut/liver barriers, enhances tight junctions
IL-35 [188,189,190]	T Effector Cells, Naïve T Cells	Stops effector T-cell proliferation, Drives the creation of new IL-35-producing regulatory cells	Spreads regulatory properties to T conv, sustaining suppression even after the original Tregs decline

IL, interleukin; TGF-β, transforming growth factor beta; APCs, antigen-presenting cells; T conv, conventional T cells; Tregs, regulatory T cells; IFN-γ, interferon gamma; Th, T helper cells; GVHD, graft-versus-host disease; MHC, major histocompatibility complex.

**Table 4:** Differentiating the mechanisms and regulation of GVHD and GVL.

Feature	Graft-Versus-Host Disease (GVHD)	Graft-Versus-Leukemia (GVL)
Core Process [205,208]	A harmful, paradoxical immune reaction against host tissues	The beneficial, desired anti-tumor immune response
Primary Immune Driver [158,159,212,213]	Donor alloreactive T cells	Donor alloreactive T cells, particularly potent CD8^+^ cytotoxic T cells
Cellular Target [158,159,167,212,213]	Healthy host tissues (e.g., gut, skin, liver) expressing alloantigens	Host malignant (leukemia) cells expressing alloantigens
Key T-Cell Mechanism [158,159,167,212,213,215]	Excessive proliferation of alloreactive effector T cells, leading to widespread tissue damage	Targeted cytotoxicity by CD8^+^ T cells, leading to leukemia clearance
Clinical Outcome [139,140,214]	A leading cause of morbidity and mortality	The primary curative mechanism of allo-HSCT
Impact of SCFA-Stabilized Tregs [213,215,216]	Tregs inhibit the widespread proliferative burst of effector T cells that drives tissue damage	Tregs do not compromise the inherent cytotoxic capacity required for the anti-leukemia effect

GVHD, graft-versus-host disease; GVL, graft-versus-leukemia; HSCT, hematopoietic stem cell transplantation; CFAs, short-chain fatty acids; CD8^+^, cluster of differentiation 8-positive T cells.

**Table 5:** Relative functional potential of key prebiotic fibers in modulating the microbiome–Treg axis [19,23,26,39,40,43,44].

Prebotic Fiber	SCFA Production	Microbiota Modulation	Barrier Effect	Treg Induction	Overall Functional Potential
Inulin/FOS	+++	+++	++	+++	High
Resistant starch	+++ (butyrate)	++	+++	+++	High
β-glucan	++	++	++	++	Moderate–high
Pectin	++ (acetate)	++	+++	++	Moderate–high
GOS	++	+++	+	++	Moderate
Arabinoxylans	++	++	++	+	Moderate
Cellulose	+	+	++	+	Low–moderate

SCFAs, short-chain fatty acids; Tregs, regulatory T cells; +, low effect; ++, moderate effect; +++, strong effect.

**Table 6:** Synergistic therapeutic strategies for GVHD modulation.

Therapeutic Strategy	Primary Mechanism of Action	Key Limitation/Challenge	Synergistic Role of Dietary Fiber (DF) & SCFAs	Combined Therapeutic Goal
Adoptive Treg Transfer [78,249,256,257,259,261,262,264,265,306,313]	Infusion of ex vivo expanded Tregs to re-establish immune tolerance.	Functional Instability: Transferred Tregs (especially iTregs) may fail to fully demethylate the Foxp3 locus and can convert into pathogenic effector cells in the inflammatory post-HSCT environment.	Ex vivo Metabolic Preconditioning: SCFAs (e.g., butyrate) are used during expansion to act as HDAC inhibitors, epigenetically “locking in” Foxp3 expression and creating more stable, resilient Tregs In vivo Support: A high-fiber diet creates an SCFA-rich gut environment that supports the persistence and function of the transferred cells.	To infuse a highly stable and persistent therapeutic Treg population that is resilient to inflammatory conversion.
LD IL-2 Therapy [78,179,225,270,273,274,290,291,292].	Provides a key homeostatic survival signal to preferentially activate and drive the numerical expansion of endogenous Tregs, which express the high-affinity IL-2 receptor (CD25).	Functional Quality: Numerical expansion alone does not guarantee the functional stability or metabolic fitness of the expanded Tregs, especially at the site of inflammation.	Metabolic & Epigenetic Stabilization: While IL-2 provides the proliferation signal, fiber-derived SCFAs provide the crucial metabolic and epigenetic support (via HDAC inhibition and mTOR modulation) required for the newly expanded Tregs to be durably functional.	To achieve both robust numerical expansion (from IL-2) and high functional/metabolic stability (from SCFAs) in the endogenous Treg compartment.
Engineered CAR-Tregs [245,271,300,301,302,303,304,305,306,307,308,309,310]	Provides “living precision medicine”. Tregs are engineered with Chimeric Antigen Receptors (CARs) to target specific alloantigens (e.g., HLA) for highly localized suppression at GVHD sites.	Cellular Exhaustion & Persistence: CAR-Tregs are at risk of dysfunction from tonic signaling. They still require metabolic support to maintain their lineage stability and persist in the inflamed gut.	Metabolic Arming: SCFAs are used during ex vivo manufacturing to reinforce Treg lineage stability via HDAC inhibition, creating a “Super-Treg” product with enhanced in vivo persistence and function.	To combine the antigen-specific precision of CARs with the metabolic and epigenetic resilience provided by SCFAs, ensuring durable, targeted suppression.

GVHD, graft-versus-host disease; HSCT, hematopoietic stem cell transplantation; Tregs, regulatory T cells; iTregs, induced regulatory T cells; CAR-Tregs, chimeric antigen receptor regulatory T cells; DF, dietary fiber; SCFAs, short-chain fatty acids; HDAC, histone deacetylase; mTOR, mechanistic target of rapamycin.

**Table 7:** Key models describing the influence of the gut microbiome on transplant outcomes.

Model	Key Mechanism	Impact on GVHD	Impact on GVL	Evidence Base
Dysbiosis model	Loss of microbial diversity and expansion of pathobionts (e.g., Enterococcus)	Increased GVHD severity via inflammation and barrier disruption	Neutral or unclear	[10,349,350,351,363]
SCFA deficiency model	Reduced production of butyrate and propionate due to low fiber intake	Increased GVHD via impaired Treg induction and epithelial dysfunction	Preserved GVL	[13,78,198]
Barrier disruption model	Epithelial injury and microbial translocation (DAMPs, LPS)	Increased cytokine release and immune activation	Indirect effect	[155,168,169]
Treg modulation model	Microbiota-derived metabolites (SCFAs, PSA) promote Treg differentiation	Reduced GVHD via immune tolerance restoration	Preserved GVL	[78,102,103,198]
FMT restoration model	Reconstitution of microbial diversity and ecosystem stability	Reduced steroid-refractory GVHD	Unknown/under investigation	[322,323,324]
Dietary fiber–microbiome model	Fermentation of dietary fiber leading to SCFA production and immune modulation	Reduced GVHD via barrier and immune effects	Preserved GVL	[19,23,202,327]

GVHD, graft-versus-host disease; GVL, graft-versus-leukemia effect; SCFAs, short-chain fatty acids; Tregs, regulatory T cells; FMT, fecal microbiota transplantation; DAMPs, damage-associated molecular patterns; LPS, lipopolysaccharide.

## Data Availability

No new data were created or analyzed in this study. Data sharing is not applicable to this article.

## Abbreviations

The following abbreviations are used in this manuscript:

ACT	Adoptive cell therapy
ALDH1A	Aldehyde dehydrogenase 1A
allo-HSCT	allogenic hematopoietic stem cell transplantation
APC	Antigen-presenting cell
aGVHD	Acute GVHD
Bas	bile acids
CAR	Chimeric antigen receptor
CCR	C-C chemokine receptor type
CNS	Conserved non-coding sequences
COX	Cyclooxygenase
CSGG	cell surface β-glucan/galactan polysaccharides
CTLA-4	Cytotoxic T-lymphocyte antigen 4
cGVHD	Chronic GVHD
cTreg	Colon Treg
CXCR	C-X-C motif chemokine receptor
DAMPs	Damage-associated molecular patterns
DC	Dendritic cell
DF	dietary fibers
EN	Enteral nutrition
FOXP3	Forkhead box p3
FMT	fecal microbiota transplantation
FOS	Fructooligosaccharides
GALT	Gut-associated lymphoid tissue
GF	Germ-free
GMP	Good Manufacturing Practice
GM	gut microbiome
GOS	Galacto-oligosaccharides
GI	Gastrointestinal
GPCRs	G-protein coupled receptors
GVHD	Graft-versus-host disease
GVL	Graft-versus-leukemia
HAT	Histone acetyltransferase
HDACs	Histone deacetylase
HIF	Hypoxia-inducible factor
IBD	Inflammatory bowel disease
IDF	Insoluble DF
IECs	Intestinal epithelial cells
IFN-γ	Interferon gamma
iTregs	inducible Tregs
LAP	LC3-associated phagocytosis
LD	Low-dose
LPS	Lipopolysaccharides
MACs	Microbiota-accessible carbohydrates
MAPK	Mitogen-activated protein kinase
MD	Mediterranean diet
MGCP	mannan/β-1,6-glucan-containing polysaccharides
MHC	major histocompatibility complex
MS	Multiple sclerosis
mTOR	Mammalian target of rapamycin
NF-Κb	Nuclear factor kappa B
NLRP	NOD-like receptor protein
NO	Nitric oxide
OS	overall survival
OXPHOS	Oxidative phosphorylation
PD-1	Programmed cell death protein 1
PDGF	Platelet-derived growth factor
PGNs	Peptidoglycans
PKB	Protein kinase B
PSA	Polysaccharide A
pTreg	Peripherally derived Treg
RA	Retinoic acid
SCFAs	short-chain fatty acids
SDF	Soluble DF
SOT	Solid organ transplantation
SPF	Specific pathogen-free
STAT	Signal Transducer and Activator of Transcription
TBI	Total body irradiation
TCR	T cell receptor
TGF-β	Transforming growth factor beta
Th	T helper
TJs	Tight junctions
TLR	Toll-like receptor
TME	Tumor microenvironment
Tregs	regulatory T cells
TSDR	Treg-specific demethylated region
T conv	Conventional T cells
tTreg	Thymus-derived Treg
WD	Western diet
